# Impact of Steam Processing on the Physicochemical Properties and Flavor Profile of *Takifugu flavidus*: A Comprehensive Quality Evaluation

**DOI:** 10.3390/foods14091537

**Published:** 2025-04-27

**Authors:** Zhihui Liu, Xiaoting Chen, Bei Chen, Yinghong Qu, Haiyan Tang, Ruowen Wu, Kun Qiao, Yongchang Su, Nan Pan, Tingru Chen, Wenzheng Shi, Zhiyu Liu

**Affiliations:** 1 College of Food Sciences & Technology, Shanghai Ocean University, Shanghai 201306, China; 2 Key Laboratory of Cultivation and High-Value Utilization of Marine Organisms in Fujian Province, National Research and Development Center for Marine Fish Processing (Xiamen), Fisheries Research Institute of Fujian, No. 7, Haishan Road, Huli District, Xiamen 361013, China; 3 College of Chemical Engineering, HUA QIAO University, Xiamen 361021, China

**Keywords:** *Takifugu flavidus*, steaming, quality, flavor characteristics, headspace gas chromatography-ion mobility spectrometry

## Abstract

As a culturally iconic Chinese delicacy, pufferfish lacks systematic research on thermal processing optimization and pre-cooked meal development, limiting its industrial standardization and quality preservation. This study aimed to bridge this gap by evaluating steaming effects on *Takifugu flavidus* quality. This study systematically evaluated its physicochemical properties and flavor profiles under different steaming durations by determining the water loss rate, mass loss rate, water distribution status, textural properties, color, and free amino acid content using an electronic nose, electronic tongue, and headspace gas chromatography-ion mobility spectrometry (HS-GC-IMS). The results indicated that the core temperature of the fish meat reached 70 °C after 9 min of steaming. With higher steaming time, its mass loss rate and water loss rate generally increased, though the water loss rate temporarily decreased at 10 min. The mass loss rate stabilized after 12.5 min. The hardness and chewiness of the fish meat increased significantly when steamed for 12.5 min or longer. After 5 min of steaming, the brightness value and yellow-blue value of the fish meat significantly increased, whereas the red-green value significantly decreased. The total free amino acid content showed a fluctuating upward trend and electronic tongue analysis revealed an increase in umami and richness after steaming. Electronic nose and HS-GC-IMS analyses demonstrated that the variety and content of volatile flavor compounds significantly increased with prolonged steaming. Sensory evaluation showed that the 10 min steaming group exhibited better texture and color, while the 15 min steaming group had the best odor. Therefore, the optimal steaming time for *T. flavidus* was determined to be 10–15 min. For home cooking, a 15 min steaming process achieves the peak abundance of flavor compounds and the highest sensory evaluation score. For the industrial production of pre-cooked meals, a 10 min steaming process can meet the doneness requirements while maintaining suitable textural properties and color stability. The findings of this study not only advance the scientific understanding of thermal processing effects on pufferfish quality attributes, but also establish a critical technological foundation for developing standardized industrial processing protocols and high-quality pre-prepared pufferfish products.

## 1. Introduction

Pufferfish has a considerable economic value and unique flavor, making it a prominent species in the aquaculture industry in China [1]. Owing to their delicate texture and exquisite taste, pufferfish are considered one of China’s most iconic culinary ingredients [2]. The main cultured pufferfish species in China are *Takifugu rubripes*, *T. obscurus*, *T. bimaculatus*, and *T. flavidus*. *T. flavidus*, a temperate demersal fish, is prized for its tender flesh, rich flavor, and high nutritional value. Chen et al. [3] analyzed the nutritional composition of four cultured pufferfish species, *T. flavidus* stands out with a crude protein content of 20.84% and a calcium content of 1737.47 mg/100 g, while its crude fat content is remarkably low at 0.24%. Additionally, *T. flavidus* exhibits the highest ∑EAA/∑NEAA (Sum of Essential Amino Acids/Sum of Non-Essential Amino Acids) ratio among the four species, thus indicating its superior nutritional profile. Its consumption is associated with health benefits such as enhanced immunity and improved gastrointestinal function, making it highly favored by consumers.

Thermal processing methods play a decisive role in determining the quality and flavor complexity of aquatic products. Thermal treatment is a pivotal step in enhancing food quality, as it initiates a cascade of intricate biochemical and chemical reactions within the food matrix. These reactions are primarily driven by factors such as elevated temperature, oxygen exposure, and lipid incorporation, which collectively influence the final flavor and texture of pufferfish [4]. Common thermal processing techniques include steaming, boiling, frying, and microwave heating. Among these, steaming is widely regarded as the healthier method, as it not only effectively preserves the nutritional components and inherent flavor characteristics of aquatic products, but also reduces the intake of oils and salts, aligning with modern dietary health requirements [5].

The temperature and duration of steaming significantly influence the quality and flavor of aquatic products. Dong et al. [6] in their study demonstrated that the texture of Pacific white shrimp varies markedly under different steaming times, with the loss of free water directly contributing to changes in shrimp meat texture. Similarly, Zheng et al. [7] observed that the water loss rate and pH of carp (*Cyprinus carpio*) muscle tissue increased with prolonged steaming time, accompanied by a noticeable expansion of the inter-fiber spaces in fish meat. These findings indicate that the steaming time not only affects the physical properties of aquatic products but may also alter their microstructural organization. Furthermore, the flavor changes in aquatic products during steaming have garnered considerable attention. Cui et al. [8] investigated the variations in flavor compounds of large yellow croaker during steaming and found that the most complex flavor profiles were achieved at 70 °C and 80 °C. Fang et al. [9] also reported significant changes in the volatile components of grass carp (*Ctenopharyngodon idella*) during steaming. Collectively, these past studies underscore the profound influence of steaming conditions on the quality and flavor of aquatic products.

Despite the growing interest in thermal processing of aquatic products, there is limited research on the effects of steaming time—a critical parameter—on the textural properties, water distribution, and volatile flavor compounds of *T. flavidus*. Therefore, this study aimed to investigate the effects of steaming on the physicochemical properties and flavor characteristics of *T. flavidus*, with the goal of identifying the optimal thermal processing conditions and providing valuable theoretical insights into the advanced processing of *T. flavidus* and the development of pre-prepared pufferfish dishes.

## 2. Materials and Methods

### 2.1. Materials

Fresh *T. flavidus* with an average weight of 300–400 g was purchased from Futian Town, Zhangpu County, Zhangzhou City, Fujian Province, China.

Methanol (chromatography grade) and trichloroacetic acid (analytical grade) were obtained from Sinopharm Chemical Reagent Co., Ltd. (Shanghai, China). Disodium hydrogen phosphate, acetonitrile (both chromatographic grade), and a series of n-ketones (2-butanone, 2-pentanone, 2-hexanone, 2-heptanone, 2-octanone, and 2-nonanone; all analytical grade) were purchased from Aladdin Biochemical Technology Co., Ltd. (Shanghai, China). MXT-WAX capillary column (30 m × 0.53 mm, 1.0 μm) (Restek Corporation, PA, USA).

### 2.2. Preparation of the Sample

Fresh *T. flavidus* were gutted and the heads and viscera were removed. The fish were then placed in perforated stainless-steel trays and steamed in a commercial steam cooker for 2.5, 5, 7.5, 10, 12.5, or 15 min. After steaming, the fish were cooled to room temperature and cut into uniform portions measuring 4 cm × 4 cm × 2 cm. Fresh fish portions (0 min) were used as the controls.

### 2.3. Core Temperature

The core temperatures of the *T. flavidus* samples were measured using a multichannel temperature tester. The temperature probe was inserted into the geometric center of the fish block, and readings were recorded every 30 s. After 15 min, the probe was removed and the temperature rise curve of the fish meat was plotted in order to analyze the heating profile during steaming.

### 2.4. Water Loss Rate

The moisture content was determined using a direct drying method in accordance with GB 5009.3-2016 [10].

(1) $$\mathrm{Water}\;\mathrm{Loss}\;\mathrm{Rate}\%=\frac{B_{1}{-B}_{2}}{B_{1}}\;\times \;100$$

B_1_: Moisture content of the unsteamed sample (g/100 g).

B_2_: Moisture content of the steamed sample (g/100 g).

### 2.5. Mass Loss Rate

Four fresh *T. flavidus* specimens of similar size were selected, weighed, and recorded as m_1_. After the steaming process, the samples were weighed again and recorded as m_2_.

(2) $$\mathrm{Mass}\;\mathrm{Loss}\;\mathrm{Rate}\%=\frac{m_{1}{-m}_{2}}{m_{1}}\;\times \;100$$

### 2.6. Water Distribution and Migration

The method was as described by Ge et al. [11] with slight modifications. The analysis was performed using MesoMR(Shanghai Niumag Electronic Technology Co., Ltd., Shanghai, China) low field nuclear magnetic resonance (LF-NMR) analyzer. The samples were placed at the center of the coil, and the transverse relaxation time (T_2_) was obtained through NMR relaxation time inversion fitting. The Carr–Purcell–Meiboom–Gill (CPMG) pulse sequence parameters were set as follows: SF (21 MHz) SW, (100 kHz) TE, (3500 ms) TD, (320,026) NS, (16 scans), and NECH (8000 echoes).

### 2.7. Texture Properties

A TA-XTplus texture analyzer was used in AMORS mode, following the method described by Liu [12], with minor modifications. A cylindrical probe (A/MBL) was used with the following parameters: pretest speed (2 mm/s); test speed (1 mm/s); post-test speed (1 mm/s); compression ratio (30%); trigger force (10 g); and time interval (5 s). Six replicates were analyzed for each sample.

### 2.8. Color

Fish block samples were cut into cubes, and the luminance values L*, red-green values a* and yellow-blue values b* of the fish samples were determined using a CR8 colorimeter.

### 2.9. Free Amnio Acid Analysis

The method of Li et al. [13] was modified. Appropriately 0.5 g of the fish sample was homogenized with 20 mL of ultrapure water, followed by the addition of 20 mL of 5% trichloroacetic acid solution. The mixture was thoroughly mixed and allowed to stand at 4 °C for 12 h. After filtration was performed, the filtrate was diluted to 50 mL, shaken, and passed through a 0.22 µm membrane filter. The filtrate was analyzed using an SL-8900 amino acid autoanalyzer(Hitachi High-Tech Corporation, Tokyo, Japan).

### 2.10. Electronic Nose Analysis

Odor analysis was performed using a PEN3.5 Electronic nose (AIRSENSE Analytics GmbH, Schwerin, Germany), following the method described by Pan et al. [14] with minor modifications. The minced fish sample (5 g) was placed in a headspace vial, sealed, and equilibrated at room temperature for 1 h. The PEN3.5 electronic nose was used for detection, and data were collected and processed using Winmuster V2.0 software. The performance characteristics of the sensors in the PEN3.5 electronic nose are presented in Table 1.

### 2.11. Electronic Tongue Analysis

Taste analysis was performed using a TS-5000Z electronic tongue(Insent Inc.,Kanagawa, Japan) following the method described by Chen et al. [15], with slight modifications. We weighed 70 g of the minced fish sample, added distilled water at a 1:3 (g/mL) ratio, and allowed it to stand at room temperature for 30 min. After centrifuging at 11,180× *g* for 10 min (4 °C), we filtered the supernatant. Thirty milliliters of the filtrate were transferred to an electronic tongue sample cup and analyzed at room temperature. Each group included four replicates, and the last three measurements were used for taste profile analysis.

### 2.12. HS-GC-IMS Analysis

The volatile flavor substances in *T. flavidus* during steaming were analyzed according to the method described by Xu et al. [16] with slight modifications. For sample preparation, accurately weighed sample (3 g) was placed in a 20 mL headspace glass vial, incubated at 60 °C for 20 min, and then injected. Each sample was measured in triplicates. The headspace conditions were as follows: incubation temperature (60 °C), incubation time (20 min), injection volume (500 µL), splitless mode, incubation speed (500 rpm), and injection needle temperature (85 °C). The GC conditions included a column temperature (60 °C), carrier gas (high-purity nitrogen, ≥99.999%), and pressure program: initial flow rate (2.0 mL/min for 2 min), linearly increased to 10.0 mL/min in 8 min, then to 100.0 mL/min in 10 min, and held for 20 min. The total run time was 40 min, and the injector temperature was 80 °C, as shown in Table 2. The IMS conditions were as follows: ionization source (tritium, 3H), drift tube length (53 mm), electric field (500 V/cm), drift tube temperature (45 °C), drift gas (high-purity nitrogen, ≥99.999%), flow rate (75.0 mL/min), and positive ion mode.

### 2.13. Sensory Evaluation

Ten trained sensory panelists evaluated *T. flavidus* samples subjected to different steaming times. The panelists assigned comprehensive scores and the highest and lowest scores were excluded from the calculation of the average score. The method followed by Zhang et al. [17], with slight modifications, was used. Table 3 outlines the sensory evaluation criteria.

### 2.14. Data Analysis

Data were analyzed using SPSS 25.0 (IBM, Armonk, NY, USA). One-way analysis of variance (ANOVA) was performed for multiple comparisons, with *p* < 0.05 considered statistically significant. Results are expressed as mean ± standard deviation, and different uppercase (A–G) and lowercase (a–g) letters or alphanumeric symbols (α–η) indicate significant differences between groups. Figures were generated using the Origin 2018 software. Volatile flavor compounds in the fish samples were analyzed using the Laboratory Analytical Viewer (LAV) 2.x (G.A.S. Company for Analytical Sensor Systems, HE, Germany) and GC-IMS Library Search software VOCal 2.x (G.A.S. Company for Analytical Sensor Systems, HE, Germany) data processing software was used to generate 3D spectra, 2D spectra, difference spectra, fingerprint plots, and PCA (Principal Component Analysis) plots via the Reporter, Gallery Plot, and Dynamic PCA modules, respectively, for the comparative analysis of volatile organic compounds between the samples.

## 3. Results and Discussion

### 3.1. Changes in Core Temperature

**Figure 1:**
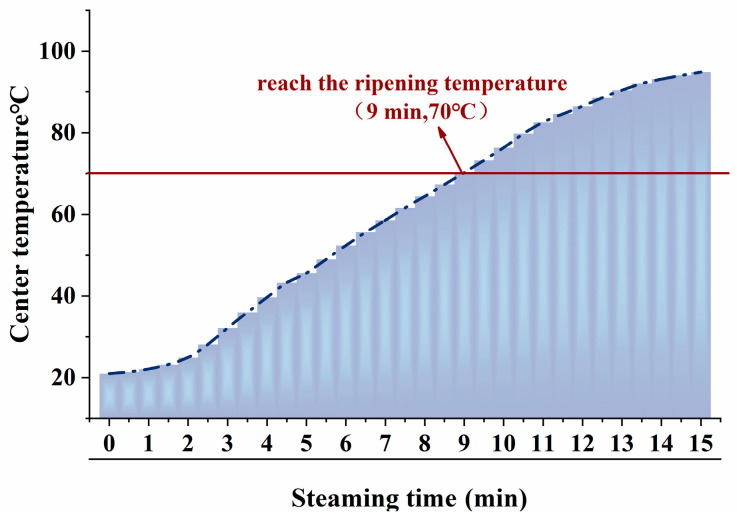
Center temperature changes in pufferfish meat at the different steaming durations.

During the steaming process, the core temperature of the fish reflects the degree of heat exchange between the steam and the fish, which is closely related to the quality characteristics and flavor changes of the fish. According to the national standard GB 31654-2021, the core temperature of food requiring thorough cooking should reach above 70 °C during processing [18]. As shown in Figure 1, during steaming, the core temperature of the fish gradually increased over time. High-temperature steam transfers heat from the exterior to the interior through thermal convection, but the initial increase in the core temperature is slow [19]. As the steaming time increases, heat continues to transfer to the interior, and the core temperature rises rapidly, reaching 70 °C at 9 min.

### 3.2. Changes in Water Loss Rate and Mass Loss Rate

**Figure 2:**
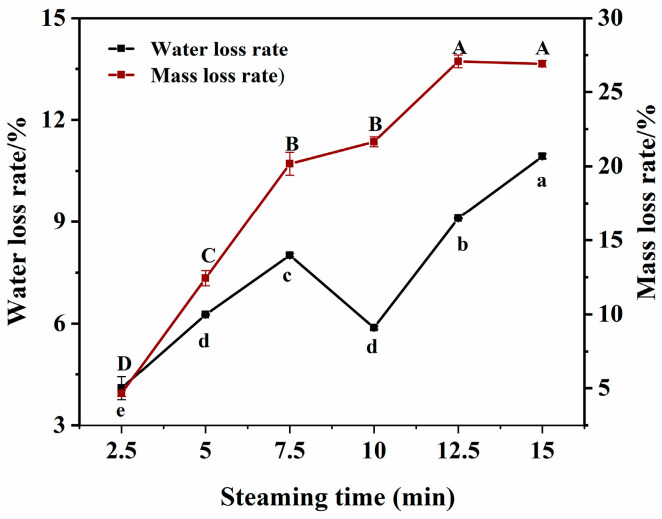
Water loss rate and weight loss rate of pufferfish meat at the different steaming durations. Different uppercase letters (A–D) indicate significant differences in the mean mass loss rate (*p* < 0.05); different lowercase letters (a–e) indicate significant differences in the mean water loss rate (*p* < 0.05).

As shown in Figure 2, both the mass loss and water loss rates of the fish increased with prolonged steaming time. During thermal processing, the activity of free water in muscle tissue intensifies, making it more easily released. Muscle fibers and connective tissues undergo contraction and stretching when heated, accompanied by juice loss. Additionally, as the heating time increased, the amount of juice lost from the muscle increased, primarily consisting of water, denatured proteins, and fat [20]. Therefore, the mass-loss rate is significantly higher than the water-loss rate. The temporary decrease in water loss rate at 10 min of steaming may be attributed to the thermal denaturation of myofibrillar proteins (60–80 °C), forming a gel network that temporarily traps water within the myofibrillar gaps [21]. As steaming continued, in the later stages (12.5–15 min), the shape of the fish blocks changed upon heating, thus reducing the distance from the center to the surface. This significantly accelerated the rate of juice loss [22].

### 3.3. Changes in Water Distribution

**Figure 3:**
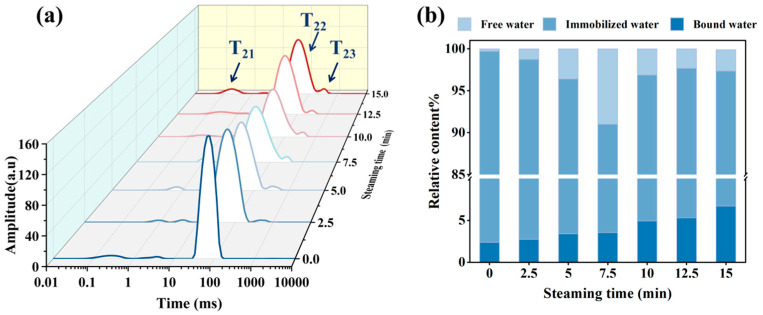
Effects of different steaming times on water distribution in pufferfish meat. LF-NMR curve (**a**) and relative content of three water states (**b**) in pufferfish meat under different steaming times.

Figure 3 shows the T_2_ peak area percentages and transverse relaxation time curves of pufferfish meat at different steaming times. As shown in Figure 3a, within the relaxation time range of 0.1–10,000 ms, three relaxation peaks are observed, corresponding to three states of water in the fish: T_21_ (1–10 ms) for bound water, T_22_ (10–100 ms) for immobilized water, and T_23_ (100–1000 ms) for free water. As the steaming time increased, the T_2_ curve shifted leftward and the relaxation time decreased. Shortening of the relaxation time reflects tighter binding between the material and water molecules, reduced water mobility, and weakened fluidity [23].

Figure 3b shows that immobilized water was the most abundant form of water in the fish, with fresh pufferfish having the highest content of immobilized water. This indicates that the muscle tissue structure of fresh fish is more compact, and that proteins have a strong water-holding capacity [24]. The relative content of bound water in the fish is low (2.38–6.70%) and gradually increases with steaming time. During thermal processing, protein denaturation leads to molecular unfolding, exposing buried hydrophobic domains and polar functional groups. These newly accessible moieties facilitate enhanced hydration through hydrogen bonding and electrostatic interactions with water molecules, predominantly via oxygen atoms, thereby increasing bound water content within the muscle [25]. The relative immobilized water content initially decreased and then increased, reaching its lowest value of 87.47% after 7.5 min of steaming, whereas the free water content first increased and then decreased, peaking at 7.5 min. This may be due to the denaturation and contraction of the muscle proteins during heating, which releases immobilized water and converts it into free water. Continued heating further exacerbates water loss from muscle tissue [26], leading to a subsequent decrease in free water content.

### 3.4. Changes in Textural Properties

**Figure 4:**
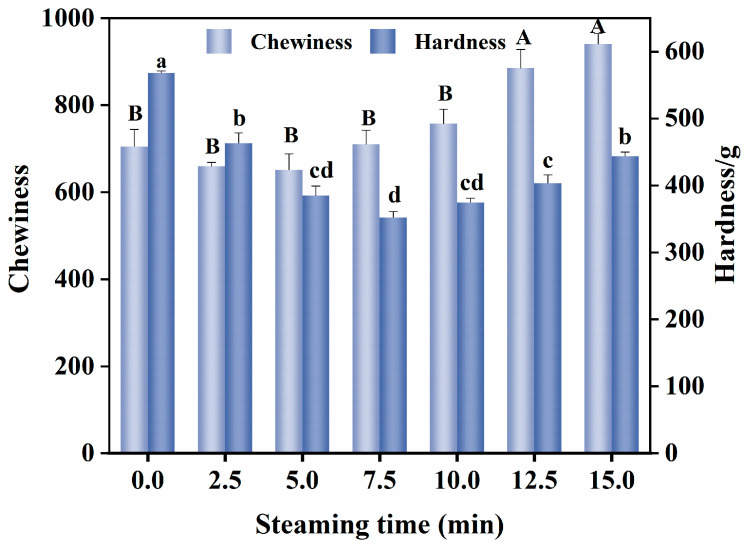
Effect of the different steaming durations on the texture of pufferfish meat. Different uppercase letters indicate significant differences in mean chewiness (*p* < 0.05); different lowercase letters indicate significant differences in hardness (*p* < 0.05).

Textural properties are key indicators for evaluating fish quality and are primarily influenced by water state and protein structure [27,28]. As shown in Figure 4, the hardness and chewiness of the fish first decreased and then increased with prolonged heating. The findings are consistent with those of Zhao et al. [24] regarding the changes in hardness and chewiness during the steaming process of sea bass muscle. During the steaming period of 0–7.5 min, the hardness of the fish decreased with increasing heating time; however, no significant difference in chewiness was observed. After steaming for 5 min, high-temperature steam was transferred from the surface to the center, causing the core temperature to increase and disrupt the muscle fiber structure. Simultaneously, the collagen in the connective tissue degrades [29], reducing its hardness to 66.79% of its initial value. When the steaming time was extended to 10 min or longer, the hardness and chewiness of the fish increased significantly (*p* < 0.05). This may be attributed to the combined effects of thermal denaturation of muscle proteins, contraction of connective tissue, and dehydration of actomyosin [30].

### 3.5. Changes in Color

**Figure 5:**
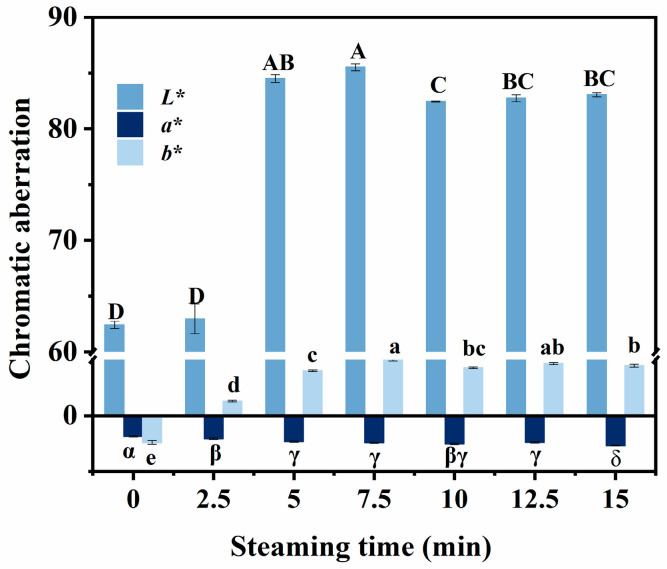
Effect of the different steaming durations on the color of pufferfish meat. Different uppercase letters (A–D) indicate significant differences in L* values (*p* < 0.05); different lowercase letters (a–e) indicate significant differences in b* values (*p* < 0.05); different alphanumeric symbols (α–δ) indicate significant differences in a* values (*p* < 0.05).

As shown in Figure 5, when compared to the control group, the L* and b* values of the steamed samples increased significantly *(p* < 0.05), whereas the a* value decreased significantly (*p* < 0.05), these findings are consistent with the study by Ye et al. [31] on color changes in mud carp fillets under different cooking methods, where steaming was observed to significantly increase L* and b* values while reducing a* values. In fresh fish, myoglobin exists primarily in the form of oxymyoglobin, which has a bright red color. During heating, high temperatures weaken the binding force between heme and the proteins in myoglobin, making it easier for heme to separate from the protein. This separation increases the exposure of heme to oxygen, thereby accelerating the oxidation process [32]. Oxidized heme no longer appears red but forms other lighter-colored compounds, thus causing the fish meat to turn white and the brightness to increase. Consequently, the L* and b* values significantly increased (*p* < 0.05), whereas the a* value significantly decreased (*p* < 0.05). As shown in Figure 5, L* reached its maximum at 7.5 min of steaming. During the later stages of steaming (7.5–15 min), the L* value was stabilized. This is because in the initial stages of steaming, protein denaturation causes water loss and the opening of muscle fibers scatters light, thereby increasing the brightness of the fish meat. Subsequently, although the steaming time was further extended, the L* value showed only a slight decrease, indicating that continued steaming had a limited effect on the brightness of fish meat, the phenomenon may result from prolonged steaming inducing further denaturation and aggregation of myofibrillar proteins, which forms a more compact network structure that could slightly enhance light scattering, thereby leading to a marginal decrease in L* values [33].

### 3.6. Changes in Free Amino Acids

**Figure 6:**
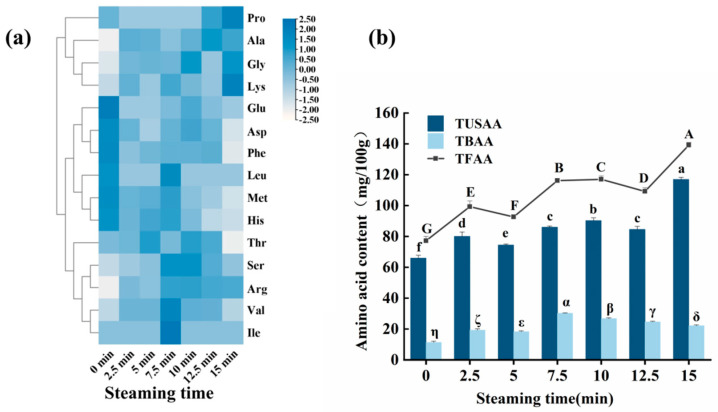
Effect of steaming duration on the content of free amino acids in puffer fish meat. Heatmap of 16 free amino acids under different steaming times (**a**) and changes in TFAA, TUSAA, and TBAA (**b**). TUSAA represents the total content of umami and sweet amino acids; TBAA denotes the total content of bitter amino acids; TFAA stands for the total free amino acid content. Different uppercase letters (A–G) indicate significant differences in TFAA content (*p* < 0.05); different lowercase letters (a–f) indicate significant differences in TUSAA content (*p* < 0.05); different alphanumeric symbols (α–η) indicate significant differences in TBAA content (*p* < 0.05).

Free amino acids (FAAs) are important flavor components in fish and are capable of interacting with other flavor substances. In addition, free amino acids serve as precursors of various flavor compounds [34]. As shown in Table S1 (See Supplementary Table S1 for detailed data), 16 amino acids were detected in the fish samples (Figure 6a). Based on their structural characteristics, these amino acids are primarily categorized as umami, sweet, or bitter amino acids. The higher the content of flavor-active amino acids, the more intense and complex the flavor of the fish meat [35]. As shown in Figure 6b, steaming significantly affected the free amino acids in the fish meat. After steaming, the total content of free amino acids, as well as the content of umami and sweet amino acids, increased.

During thermal processing, partial hydrolysis of fish meat proteins may occur, releasing free amino acids. Therefore, as the steaming time increases, the overall free amino acid content increases [26]. However, temporary decreases are observed at 5 min and 12.5 min. In the initial stages of steaming, the thermal activation of endogenous proteases in fish meat accelerates the breakdown of myofibrillar proteins and collagen into small peptides and free amino acids, leading to a rapid increase in their content, particularly of umami and sweet amino acids [36,37]. After 5 min of steaming, when the temperature exceeds the optimal range for enzyme activity, proteases may be inactivated due to thermal denaturation, temporarily slowing the hydrolysis rate and causing a deceleration or even a brief decline in the increase in free amino acids [38]. At 12.5 min, the decrease in free amino acids may be attributed to their participation in Maillard reactions or Strecker degradation, where they combine with reducing sugars to form volatile flavor compounds, such as pyrazines or aldehydes. The consumption rate exceeds the accumulation rate, resulting in a decline in the free amino acid content [39]. The total bitter amino acid content initially increases and then decreases during steaming. Significant accumulation of bitter amino acids (e.g., isoleucine, leucine, and valine) was observed after 5–7.5 min of steaming. In the later stages of steaming (10–15 min), the bitter amino acid content gradually decreased, indicating that prolonged steaming reduced bitterness and improved the overall taste.

Lysine was present in the highest concentration in raw *T. flavidus* meat, followed by glycine, alanine, threonine, arginine, and histidine. After steaming was performed, the levels of lysine, alanine, glycine, and arginine increased significantly, while the levels of bitter amino acids such as methionine, histidine, and phenylalanine decreased significantly. Lysine exhibits dual characteristics of bitterness and sweetness, which can mask undesirable flavors such as bitterness, thereby improving the overall flavor of food [40]. Additionally, lysine and glutamic acid work synergistically to enhance the overall flavor profile of foods [41]. Alanine and glutamic acid, as sweet amino acids, produce a synergistic effect when coexisting, significantly enhancing the umami perception of fish meat, which not only reduces the bitterness of fish meat, but also imparts a fresh and sweet flavor. Although arginine is a bitter amino acid, it contributes to an overall pleasant taste rather than bitterness [42]. Chen and Zhang [43] demonstrated that the addition of arginine could optimize the umami taste of model solutions, while reducing sourness. These results indicate that steaming significantly enhances the umami and sweetness of fish meat, and that an appropriate steaming duration can optimize the flavor profile. Methionine, a sulfur-containing amino acid, may undergo structural degradation during high-temperature steaming, especially under prolonged heating. This can lead to oxidation or desulfurization reactions [44], resulting in a decrease in its content. Additionally, high temperatures may promote the participation of phenylalanine in Strecker degradation, such as in the formation of volatile aldehydes [45].

### 3.7. Analysis of Electronic Tongue Results

**Figure 7:**
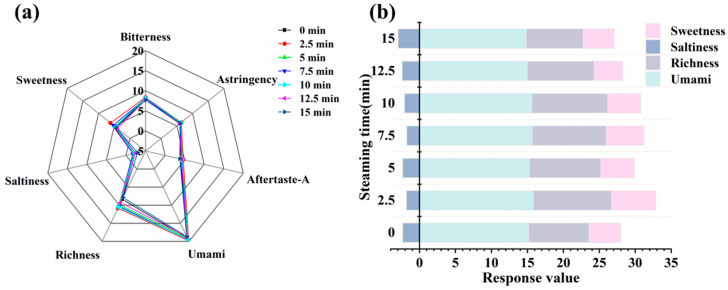
Effect of steaming duration on the electronic tongue results in the pufferfish meat. Radar chart (**a**) and Stacked chart (**b**) of electronic tongue sensor for pufferfish meat.

By simulating the human taste system, the electronic tongue qualitatively identifies and quantitatively analyzes the taste of food [46]. As shown in Figure 7, different steaming times had no significant effect on bitterness, astringency, or aftertaste, with response value differences of less than 1, indicating that humans may not perceive differences in these taste attributes owing to steaming time [47]. In contrast, significant differences were observed in the response values of umami, saltiness, sweetness, and richness at the different steaming times. Both the unsteamed and steamed groups showed high response values for the umami and richness sensors, indicating that the fish meat exhibited strong umami and richness. After steaming, the umami taste and richness of the fish meat increased. During steaming, the release and transformation of water-soluble flavor compounds (e.g., free amino acids and nucleotides) enhance the umami and richness [39]. Sweetness initially increased and subsequently decreased with prolonged steaming time. This may be due to the enzymatic breakdown of glycogen into reducing sugars during the initial stages of steaming, increasing the concentration of soluble sweet compounds, coupled with the thermal denaturation of proteins releasing free amino acids (e.g., glycine and alanine), collectively leading to a significant increase in the sweetness at 2.5 min [48].

### 3.8. Analysis of Volatile Flavor Compounds by Electronic Nose

**Figure 8:**
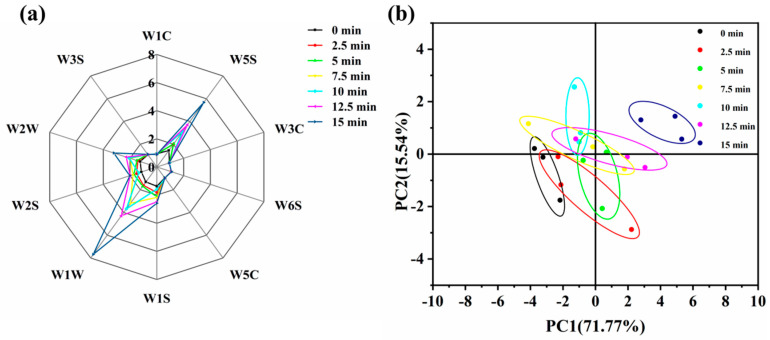
Effect of the steaming duration on the electronic nose results in pufferfish meat. Radar chart of E-nose sensor (**a**) and PCA (**b**) for puffer fish meat at different steaming durations.

The results of the e-nose radar chart are shown in Figure 8a. As the steaming time increased, the response values of the samples for sensors W5S, W1W, and W2W increased significantly, with the highest response being observed at 15 min. This indicated that prolonged steaming enhanced the contribution of nitrogen oxides, sulfides, aromatic compounds, and organosulfides to the overall aroma of the samples. When compared to the fresh samples, steaming resulted in a greater abundance of volatile flavor compounds.

Principal Component Analysis (PCA) transforms electronic nose sensor data by extracting uncorrelated feature vectors and linearly combining them to characterize the overall information of the samples [49]. PC1 (71.80%) and PC2 (15.50%) explained 87.30% of the total variance, exceeding 85.00%, indicating that these two principal components captured the main information on volatile compounds in fish meat and sufficiently represented the overall odor characteristics of the samples. As shown in Figure 8b, the PCA results for the fish meat under different steaming times revealed that the 0 min and 2.5 min samples clustered together, suggesting similar volatile flavor profiles. Additionally, the 15 min steamed sample was distinctly separated from the other samples along the PC1 axis, indicating significant differences in odor composition. This may be attributed to the prolonged steaming time, which promotes the generation of volatile flavor compounds.

### 3.9. HS-GC-IMS Analysis

**Figure 9:**
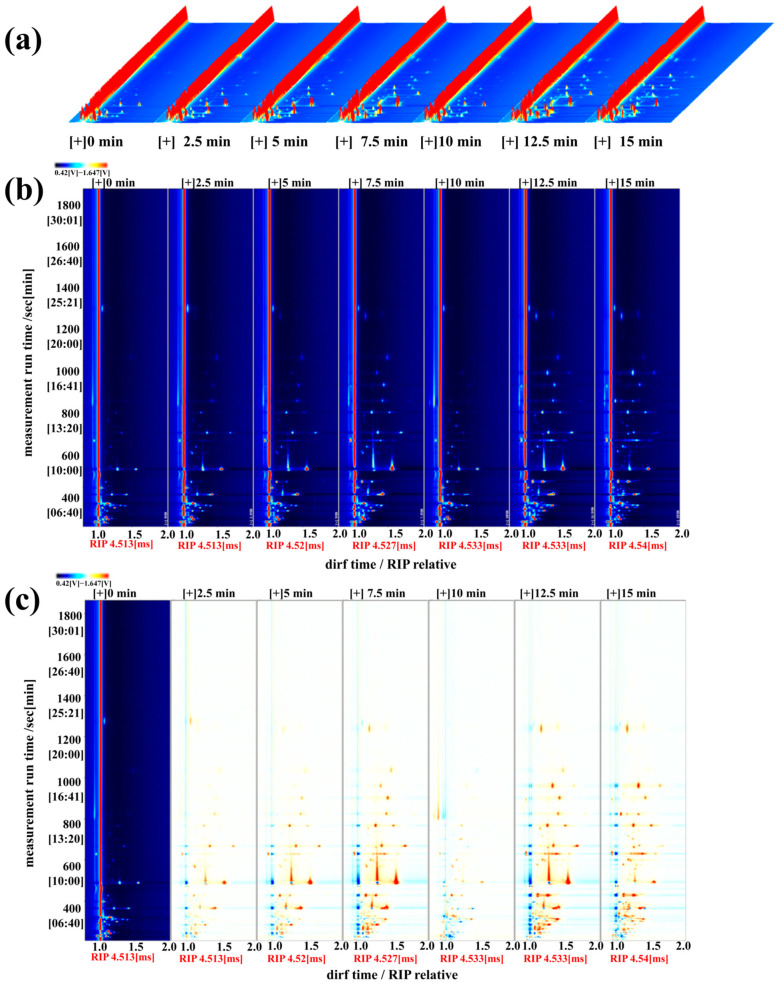
Effect of steaming duration on the volatile compounds in puffer fish meat. 3D (**a**) and 2D (**b**) gas chromatography-ion mobility spectrometry profiles and difference plot (**c**) of volatile compounds in puffer fish meat.

Figure 9 shows the gas chromatography-ion mobility spectrometry (GC-IMS) results of the volatile flavor compounds in *T. flavidus* at different steaming times. The 3D (a) and 2D (b) plots illustrate the complex compositional distributions of these compounds. Each point on either side of the RIP (Reactive Ion Peak) corresponds to a volatile compound, with the color intensity indicating the peak strength ranging from blue (low intensity) to red (high intensity) [50]. As shown in Figure 9a,b, there were notable differences in volatile organic compounds among the samples. When compared to the unsteamed sample, the steamed samples exhibited a significant increase in the number of light blue and red marked points, with the red points showing a progressively deeper color intensity. This indicates a substantial increase in volatile flavor compounds after steaming.

To visually compare the differences in volatile components between the samples, the spectra of the 0 min sample were used as a reference, and the spectra of the remaining samples were subtracted to generate difference plots (Figure 9c). In the difference plots, white areas indicate consistent volatile flavor compounds between the two samples, red areas represent higher concentrations than the reference, and blue areas indicate lower concentrations [51]. As shown in Figure 9c, the spectral plots of the six steamed fish groups display varying degrees of red points. Compared with the control, steamed fish exhibited an increase in volatile flavor compounds, which was also confirmed by the GC-IMS spectra. These results demonstrate that steaming enhances the variety and content of volatile flavor compounds in fish meat.

The volatile components in the samples were qualitatively analyzed using the GC retention index (NIST 2020) and IMS migration time databases integrated into VOCal 2.x software (G.A.S. Company for Analytical Sensor Systems, HE, Germany). Owing to the ionization zone, a single compound may generate more than one signal (monomer, dimer, or even trimer) as its concentration increases, leading to the detection of dimers of some volatile substances [52]. Table S2 lists the volatile components identified in the GC-IMS database (See Supplementary Table S2 for detailed data). A total of 55 volatile compounds were identified during the steaming of *T. flavidus*, with carbon chain lengths primarily ranging from C2 to C10. The compounds detected were mainly alcohols, aldehydes, ketones, and esters.

**Figure 10:**
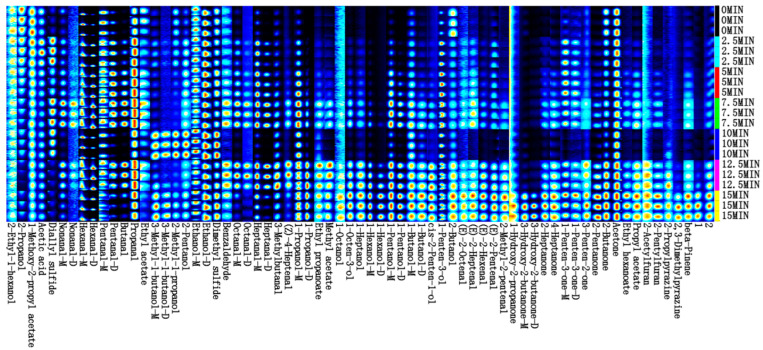
Volatile fingerprint of pufferfish meat at different steaming durations.

The changes in the volatile compounds during the steaming process were further analyzed, and the resulting flavor fingerprints are shown in Figure 10. The color depth and area size of the points reflect the relative concentration of the compounds, with darker colors and larger areas indicating higher concentrations, and vice versa [16]. As shown in Figure 10, steaming time significantly affected the signal intensity of volatile compounds in fish meat, reaffirming that steaming markedly increased the variety and content of volatile flavor compounds. The fluctuations in the types and contents of volatile flavor compounds during steaming may be closely related to chemical processes such as lipid oxidation [53]. Among the volatile compounds generated after steaming, ketones and aldehydes were predominant, which was consistent with the electronic nose analysis results.

In both the non-steamed and steamed *T. flavidus*, elevated levels of propanal (pungent, green grassy), 2-ethyl-1-hexanol (citrus, fresh floral, greasy), and acetone (acetone, fresh, sweet fruity, wine) were detected, suggesting their potential roles as characteristic flavor compounds. The non-steamed samples exhibited lower overall volatile compound concentrations; however, significantly higher levels of 2-propanol, 1-methoxy-2-propyl acetate (banana, fruity), 2-butanol, and 2-butanone (fruity, camphoraceous) were observed in comparison to the steamed group. During steaming (2.5–5 min), compounds such as diallyl sulfide, 2-pentanol, n-butanal, 1-penten-3-one, octanal, and heptanal emerged, primarily derived from the thermal degradation of proteins, lipids, and carbohydrates, as well as Maillard reactions and lipid oxidation under high-temperature conditions [45]. Notably, diallyl sulfide imparts garlic notes, whereas 2-pentanol contributes to fusel oil and green (refers to fresh, herbaceous, or slightly vegetal notes, typically associated with the scent of fresh leaves or grass.) odors. The presence of 1-penten-3-one introduced strong pungent odors and octanal-enhanced citrus or floral aromas. These findings underscore the dynamic transformation of flavor profiles during steaming driven by complex physicochemical interactions.

At 7.5 min of steaming, the contents of decanal, hexanal, pentanal, butanal, propanal, and ethyl acetate increased, which were mainly generated by lipid oxidation and the Maillard reaction [45]. Among them, decanal and hexanal contributed to the grassy and citrus flavors, pentanal enhanced the fruity and nutty notes, and butanal and propanal imparted slight pungency. Ethyl acetate imparts a fruity aroma. After 10 min of steaming, the aldehyde compounds decreased, whereas alcohols, such as 3-methyl-1-butanol (whiskey, banana, fruity), 2-methyl-1-propanol (afresh, alcoholic, leather), and 2-pentanol (fusel oil, green) significantly increased. The low rate of water loss after 10 min of steaming, which causes the aldehydes to dissolve in water or undergo thermal reduction reactions to form alcohols [54]. Additionally, the high moisture content slows lipid oxidation, reducing the formation of new aldehydes and consuming existing ones, leading to the accumulation of alcohols.

In the later stages of steaming (12.5–15 min), the alcohol content decreased, whereas those of aldehydes, ketones, pyrazines, furans, and lipids increased. This may be attributed to the dehydrogenation of alcohols to form aldehydes at high temperatures or their further oxidation to ketones [55]. Concurrently, the reduction in the moisture content accelerates the Maillard reaction, leading to the formation of pyrazine compounds (e.g., 2,3-dimethylpyrazine, which imparts a nutty flavor) through the reaction of aldehydes with amino acids [56]. This was consistent with the observed decline in free amino acids at 12.5 min. Additionally, aldehydes reacted with α-dicarbonyl compounds via Strecker degradation to produce methyl ketones (e.g., 2-pentanone, which has acetone, fresh, sweet fruity, wine aroma, and 2-butanone) and other volatile substances [54]. Aldehydes play a crucial role in the characteristic flavor of fish meat. (E)-2-heptenal (spicy, green vegetables, fresh, fatty), (E)-2-octenal (fresh cucumber, fatty, green herbal, banana, green leaf), octanal, and heptanal, which are mainly derived from the degradation of unsaturated fatty acids, are more abundant in the later stages of steaming [57]. Ketones (e.g., 2-heptanone, which has a pear, banana, fruity, and a slight medicinal fragrance aroma; 2-octanone, with creamy and mushroom notes) contribute to the mitigation of fishy odors and may modify the flavor profile of fish meat [50,58]. The synergistic action of alcohols, aldehydes, ketones, lipids, pyrazines, and furans in the later stages of steaming enhances the flavor of fish meat.

During the steaming process, the transformation of volatile compounds in fish meat, such as alcohols, aldehydes, alcohols, ketones, pyrazines, and furans, results from a dynamic equilibrium between lipid oxidation, reduction reactions, and the Maillard reaction. In the early stages of steaming (2.5–7.5 min), low moisture content promotes lipid oxidation. At the midpoint (10 min), high moisture content inhibits oxidation and promotes reduction reactions. In the later stages (12.5–15 min), the moisture content decreases again, accelerating the oxidation and condensation reactions to form a complex flavor profile. Steaming facilitates the conversion of flavor precursor compounds, enriches the flavor compound system, retains the original flavor, and enhances overall quality, whereas moderate steaming promotes the release of volatile flavor compounds [59].

### 3.10. PCA and Nearest Neighbor Distance Analysis

**Figure 11:**
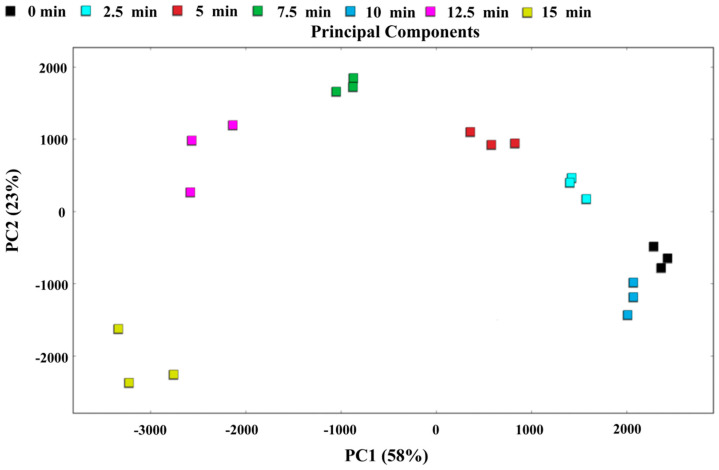
PCA score plot of volatile components in pufferfish meat at the different steaming durations.

PCA is an efficient dimensionality-reduction technique that can intuitively present differences among various samples using a PCA score plot. In this study, we conducted a systematic PCA of 55 volatile flavor compounds in *T. flavidus* samples steamed for different durations. As shown in Figure 11, the PCA plot illustrates the differences in the composition of volatile flavor compounds in fish meat at various steaming times. The results indicate that the first principal component (PC1) accounted for 58.00% of the variance, while the second principal component (PC2) accounted for 23.00%, with a cumulative contribution rate of 81.00%. This suggests that the selected principal components adequately represent most of the original variable information for the volatile compounds [55].

**Figure 12:**
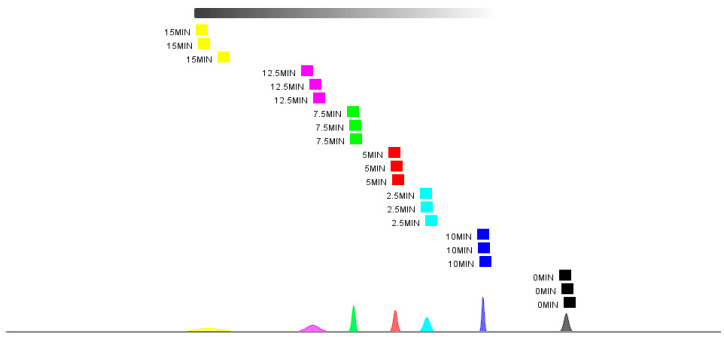
Nearest neighbor analysis plot of HS-GC-IMS for pufferfish meat at the different steaming durations.

In Figure 11, the unsteamed samples are located in the far-right region, clearly indicating significant changes in volatile organic compounds (VOCs) before and after steaming, which is highly consistent with the results obtained from the electronic nose analysis. Further analysis also revealed that as the steaming time increased, the distance between each sample group and the 0 min sample becomes greater (except for the 10 min group), indicating evident differences in the VOC content in fish meat at different steaming times. The proximity of the 10 min and 0 min samples may be attributed to the reduction of aldehydes and the increase in alcohols at 10 min. The nearest neighbor distance analysis (Figure 12) showed results consistent with those of the PCA.

### 3.11. Sensory Evaluation Results

**Figure 13:**
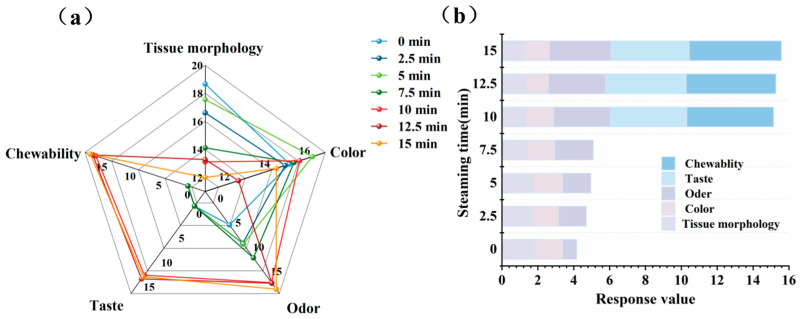
Effect of steaming duration on the sensory evaluation in puffer fish meat. Sensory radar (**a**) and stacked chart (**b**) of pufferfish meat.

Based on the evaluation criteria listed in Table 3, a comprehensive sensory assessment was conducted on fish meat samples treated for different steaming durations, covering five key dimensions: texture, color, aroma, flavor, and chewiness. The results are presented in Figure 13. It was observed that as the steaming time increased, the texture of the fish meat gradually deteriorated, while the aroma score steadily improved, which is consistent with the findings from the electronic nose analysis. Among the three groups of samples steamed for 10–15 min (samples steamed for 0–7.5 min were not included in the flavor and chewiness evaluations due to their incomplete cooking), the 10 min group exhibited the best texture and color, while the 15 min group achieved the highest aroma score, aligning with the results from the electronic nose and HS-GC-IMS analyses. At 12.5 min, the flavor was relatively good, but showed no significant difference compared to the other two groups. In terms of chewiness, an increase was noted with extended steaming times (10–15 min), which is in agreement with the textural analysis results. The overall sensory scores for the 10–15 min groups showed a gradual upward trend.

## 4. Conclusions

In this study, we systematically investigated the effects of steaming time on the quality and flavor profile of *T. flavidus*. The results demonstrated that fish reached optimal doneness after 9 min of steaming. Prolonged steaming led to increased mass loss and dehydration rates, which stabilized after 12.5 min. The relative content of immobile water exhibited a dynamic trend, initially decreasing and then increasing, whereas hardness and chewiness followed a similar pattern. After 5 min of steaming, the L^*^ and b^*^ values significantly increased, whereas a^*^ value notably decreased. The total free amino acid content fluctuated but showed an overall upward trend with extended steaming times. Electronic tongue analysis revealed enhanced umami and richness of steamed fish. The electronic nose and HS-GC-IMS results indicated a significant increase in the variety and concentration of volatile flavor compounds after steaming. Sensory evaluation highlighted that the 10 min group exhibited superior texture and color, while the 15 min group scored the highest in aroma. Comprehensive analysis determined that the optimal steaming duration was 15 min for home cooking, maximizing the flavor compound abundance and overall sensory scores, and 10 min for industrial pre-prepared dishes, ensuring doneness while maintaining desirable texture and color. This study provides a scientific foundation for the steaming process of *T. flavidus*, offering valuable theoretical and practical insights for optimizing aquatic product quality and flavor, as well as advancing pre-prepared dish production.

Nevertheless, this study has several limitations that should be acknowledged. The lack of total amino acid analysis may compromise nutritional assessment accuracy, while lab-scale conditions could differ from industrial processing parameters. Additionally, molecular mechanisms connecting protein denaturation to flavor development require further validation. To address these gaps, future research should prioritize the following: quantifying nutrients before and after steaming and correlating proteolysis with umami peptide formation; developing advanced steaming techniques through pressure-assisted methods and pilot-scale trials; and employing molecular modeling with EPR to decode protein-flavor mechanism. Such multidisciplinary approaches will effectively translate these findings into industrial applications for premium aquatic product development.

## Figures and Tables

**Table 1:** Sensors and main application in PEN3.5.

Array Number	Sensor Name	Representative Substance Categories	Performance Description
1	W1C	Aromatic	Aromatic constituents, benzene
2	W5S	Broadrange	High sensitivity and sensitive to nitrogen oxides
3	W3C	Aromatic	Sensitive aroma, ammonia
4	W6S	Hydrogen	Mainly selective for hydrides
5	W5C	Arom-aliph	Short-chain alkane aromatic component
6	W1S	Broad-methane	Sensitive to methyl
7	W1W	Sulphur-organic	Sensitive to sulfides
8	W2S	Broad-alcohol	Sensitive to alcohols, aldehydes and ketones
9	W2W	Sulph-chlor	Aromatic ingredients, sensitive to organic sulfides
10	W3S	Methane-aliph	Sensitive to long-chain alkanes

**Table 2:** Gas chromatography conditions.

Time	E1 (Drift Gas Flow Rate)	E2 (Carrier Gas Flow Rate)	R (Data Acquisition)
00:00,000	75.0 mL/min	2.0 mL/min	recording
02:00,000	75.0 mL/min	2.0 mL/min	-
10:00,000	75.0 mL/min	10.0 mL/min	-
20:00,000	75.0 mL/min	100.0 mL/min	-
40:00,000	75.0 mL/min	100.0 mL/min	stop

**Table 3:** Sensory evaluation criteria for the pufferfish at different steaming durations.

Scores	Tissue Morphology (10%)	Color (10%)	Odor (20%)	Taste (30%)	Chewiness (30%)
17–20	Maintaining the original morphology of the fish meat with a uniform and compact tissue structure	Bright and uniform color	Distinct steamed fish aroma, free from fishy or rancid odors	Strong umami characteristic of *T. flavidus*, free from bitter or fishy odors	Significant meat elasticity and chewiness, no sense of crumbling
13–16	The original morphology of the fish meat is preserved, with a relatively compact tissue structure	Bright color with good appearance	Fresh steamed aroma, free from fishy or rancid odors	Distinct umami characteristic of *T. flavidus*, free from bitter or fishy odors	Noticeable meat elasticity with moderate chewiness, no sense of crumbling
9–12	The morphology is slightly loose, with moderate uniformity and compactness of the tissue	Dull color with a slightly grayish-white appearance	Moderate aroma with slight fishy or rancid odors	Moderate umami characteristic of *T. flavidus*, with slight bitter or fishy odors	Moderate meat elasticity and chewiness, with a slight sense of crumbling
5–8	The morphology is loose, with poor tissue uniformity	Dull color with a distinctly grayish-white appearance	Weak aroma with noticeable fishy or rancid odors	Weak umami characteristic of *T. flavidus*, with noticeable bitter or fishy odors	Poor elasticity and chewiness, loose texture with a sense of crumbling
1–4	The integrity of the morphology cannot be maintained, and the tissue exhibits significant fragmentation	Uneven and grayish color with dark gray or black spots	Almost no aroma, with prominent fishy or rancid odors	No umami characteristic of *T. flavidus*, with prominent bitter or fishy odors	Low elasticity, loose texture, and a pronounced sense of crumbling

## Data Availability

The original contributions presented in the study are included in the article/Supplementary Materials; further inquiries can be directed to the corresponding author.

## Supplementary Materials

The following supporting information can be downloaded at https://www.mdpi.com/article/10.3390/foods14091537/s1. All supporting information is provided at the end of the article, Table S1: Effect of steaming duration on free amino acid content in pufferfish meat; Table S2: Volatile compound peak areas in gas chromatography-ion mobility spectrometry (GC-IMS) profiles of pufferfish meat at different steaming times.
