# Supplementary material for: Impact of Steam Processing on the Physicochemical Properties and Flavor Profile of Takifugu flavidus: A Comprehensive Quality Evaluation
Source: Foods. 2025 Apr 27;14(9):1537. doi: 10.3390/foods14091537 (PMC12071282; doi:10.3390/foods14091537)
Supplement: Supplementary file 1 [file foods-14-01537-s001.zip › foods-3574207-supplementary.pdf]

Supplementary Materials

Table S1. Effect of steaming duration on free amino acid content in pufferfish meat

Free Amino Acid	Content (mg/100g)						
	0 min	2.5 min	5 min	7.5 min	10 min	12.5 min	15 min
Glu	6.25±0.15^{a}	ND	ND	1.09±0.15^{c}	2.61±0.08^{b}	0.89±0.32^{c}	ND
Asp	1.03±0.02^{a}	0.55±0.03^{c}	0.25±0.03^{d}	0.56±0.03^{c}	0.73±0.03^{b}	0.55±0.05^{c}	ND
Gly	8.13±0.48^{d}	17.71±0.92^{b}	18.46±0.21^{b}	18.23±0.08^{b}	24.64±0.44^{a}	14.86±0.15^{c}	25.33±0.32^{a}
Ser	0.55±0.05^{d}	1.15±0.3^{c}	1.48±0.12^{c}	3.51±0.01^{a}	3.51±0.08^{a}	2.43±0.06^{b}	1.40±0.040^{c}
Ala	8.99±0.24^{e}	12.31±0.59^{abc}	12.13±0.12^{bc}	11.33±0.3^{c}	12.16±0.41^{bc}	13.44±0.33^{c}	12.81±0.13^{ab}
Thr	2.48±0.29^{c}	2.61±0.11b^{c}	3.42±0.06^{a}	2.51±0.02^{c}	3.39±0.04^{a}	3.03±0.08^{ab}	0.54±0.13^{d}
Pro	5.03±0.54^{a}	0±0	0±0	0±0	0±0	9.81±0.51^{b}	17.09±0.18^{c}
Lys	33.48±0.31^{f}	45.7±0.69^{c}	38.62±0.18^{e}	48.72±0.24^{b}	43.18±0.72^{d}	39.55±0.45^{e}	59.35±0.54^{a}
Met	1.72±0.17^{a}	0.92±0.14^{cd}	1.05±0.03^{c}	1.36±0.06^{b}	0.65±0.03^{de}	0.45±0.02^{e}	ND
Phe	1.8±0.14^{a}	1.12±0.11^{b}	1.25±0.07^{b}	1.31±0.01^{b}	1.32±0.06^{b}	1.35±0.02^{b}	0.68±0.03^{c}
Val	0.97±0.03^{c}	1.92±0.23^{b}	1.93±0.02^{b}	3.28±0.01^{a}	2.08±0.04^{b}	2.02±0.01^{b}	1.07±0.04^{c}
Arg	3.86±0.27^{d}	14.12±0.21^{c}	12.72±0.33^{c}	19.15±0.04^{a}	19.52±0.24^{a}	17.91±0.25^{b}	17.61±0.2^{b}
His	1.61±0.14^{a}	1.19±0.15^{b}	1.38±0.09a^{b}	1.46±0.07a^{b}	1.11±0.06^{bc}	0.78±0.09^{cd}	0.72±0.13^{d}
Ile	ND	ND	ND	2.11±0.1^{a}	ND	ND	ND
Leu	1.37±0.1^{b}	ND	ND	1.54±0.04^{a}	ND	ND	ND
TUSAA	65.89±1.91^{f}	80.03±2.82^{d}	74.36±0.76^{e}	85.95±0.87^{c}	90.22±1.77^{b}	84.56±1.91^{c}	116.95±1.29^{a}
TBAA	11.33±0.86^{g}	19.27±0.85^{f}	18.33±0.54^{e}	30.21±0.33^{a}	24.68±0.53^{b}	22.51±0.49^{c}	20.08±0.51^{d}
TFAA	77.22±2.77^{g}	99.3±3.67^{e}	92.69±1.30^{f}	116.16±1.20^{b}	114.9±2.30^{c}	107.07±2.41^{d}	137.03±1.79^{a}

Note: ND indicates "not detected"; TUSAA represents the total content of umami and sweet amino acids; TBAA denotes the total content of bitter amino acids; TFAA stands for the total free amino acid content.

Table S2. Volatile compound peak areas in gas chromatography-ion mobility spectrometry (GC-IMS) profiles of pufferfish meat at different steaming times.

Compound	CAS#	Formula	Rt(s)	Dt（RIPr	0 min	2.5 min	5 min	7.5 min	10 min	12.5 min	15 min
**1-Octanol**	**C111875**	C8H18O	1795.69	1.477	265.02±11.47	276.54±6.32	272.25±28.22	283.86±15.94	258.81±10.81	320.45±21.61	352.74±44.9
**Benzaldehyde**	**C100527**	C7H6O	1420.2	1.15858	118.83±5.53	104.43±6.30	127.29±1.87	175.85±6.24	67.55±6.67	172.49±1.05	117.02±23.32
**2-Acetylfuran**	**C1192627**	C6H6O2	1370.39	1.11358	94.84±6.28	109.53±2.26	109.89±4.45	117.71±4.49	102.35±5.07	178.83±8.72	177.63±16.67
**2-Ethyl-1-hexanol**	**C104767**	C8H18O	1381.22	1.42149	155.41±15.83	140.78±3.02	139.28±5.45	141.27±5.12	143.55±15.400	139.69±0.70	121.7±18.72
**Aceticacid**	**C64197**	C2H4O2	1277.28	1.06016	2473.49±150.3	3538.85±364.5	2547.43±184.04	2181.85±45.8	2460.75±41.34	2433.5±33.88	2432.45±29.72
**1-Octen-3-ol**	**C3391864**	C8H16O	1233.97	1.16561	164.87±13.46	358.72±31.05	629.5±54.61	1481.04±89.45	252.96±17.19	1915.03±131.03	2299.72±37.73
**1-Heptanol**	**C111706**	C7H16O	1246.97	1.40603	96.79±4.55	163.45±16.15	227.3±21.11	333.97±17.86	162.16±11.51	562.53±28.75	635.61±4.6
**(E)-2-Octenal**	**C2548870**	C8H14O	1115.96	1.33151	57.5±1.74	78.37±6.31	87.83±7.17	94.58±4.87	62.29±7.75	98.57±7.53	113.21±9.82
**Nonanal-M**	**C124196**	C9H18O	1035.84	1.48617	516.85±100.99	778.35±112.15	943.61±28.57	1479.54±57.27	320.28±55.27	1259.47±135.69	670.08±110.35
**Nonanal-D**	**C124196**	C9H18O	1033.68	1.94593	69.68±5.18	72.76±4.29	78.06±6.81	121.91±4.7	65.66±4.42	106.28±13.54	59.65±13.09
**1-Hexanol-M**	**C111273**	C6H14O	960.06	1.33151	193.21±7.25	351.45±7.18	431.28±56.46	1214.58±310.13	481.92±42.17	2795.71±435.2	3704.25±19.12
**1-Hexanol-D**	**C111273**	C6H14O	961.142	1.64786	140.81±16.99	130.82±4.53	134.32±2.05	195.4±27.3	131.79±12.33	573.73±153.72	968.03±27.12
**2,3-Dimethylpyrazine**	**C5910894**	C6H8N2	922.166	1.11499	41.97±4.29	47.65±2.31	45.09±7.85	53.61±3.75	49.58±2.18	71.78±4.13	195.28±22.45
**cis-2-Penten-1-ol**	**C1576950**	C5H10O	895.828	0.94505	114.12±5.88	136.08±5.12	183.91±13.56	325.74±12.07	101.56±8.22	534.12±28.01	590±16.79
**(E)-2-Heptenal**	**C18829555**	C7H12O	884.569	1.26081	25.86±2.88	42.31±5.27	72.15±7.88	100.72±7.07	24.58±2.98	85.04±4.7	108.25±5.58
**1-Hydroxy-2-propanone**	**C116096**	C3H6O2	852.901	1.07575	71.03±1.13	73.65±1.74	68.58±1.16	72.9±4.47	78.08±2.37	82±5.06	136.01±12.12
**3-Hydroxy-2-butanone-**	**C513860**	C4H8O2	822.64	1.07575	226.19±21.55	179.35±5.1	178.73±3.63	184.6±14.61	225.92±16.46	297.17±37.65	1649.27±191.2

**3-Hydroxy-2-butanone-D**	**C513860**	C4H8O2	824.047	1.33198	61.21±2.58	92.08±9.38	130.68±9.93	177.9±2.15	62.61±5.57	193.77±5.26	712.21±140.77
**Octanal-M**	**C124130**	C8H16O	825.455	1.41739	179.18±36.11	514.2±76.92	740.69±39.71	1078.17±19.17	146.75±11.34	1142.92±69.47	391.62±101.88
**Octanal-D**	**C124130**	C8H16O	825.455	1.82373	61.82±4	76.78±9.04	100.52±8.57	173.96±11.09	61.95±1.62	189.88±29.35	106.55±6.2
**1-Pentanol-M**	**C71410**	C5H12O	767.749	1.25822	184.8±38.23	591.74±28.81	1392.12±114.23	2066.41±42.91	633.81±34.6	2155.58±44.6	2358.14±8.15
**1-Pentanol-D**	**C71410**	C5H12O	767.749	1.51704	62.76±5.77	113.88±6.75	419.22±62.59	974.86±52.76	125.84±7.2	1117.9±43.02	1364.43±6.42
**(Z)-4-Heptenal**	**C6728310**	C7H12O	755.082	1.14822	63.53±5.86	85.99±5.78	121.59±9.5	263.21±7.45	77.6±4.52	387.3±30.97	160.8±20.39
**Ethylhexanoate**	**C123660**	C8H16O	743.118	1.33198	26.09±2.15	35.54±3.85	51.25±2.78	75.7±2.48	26.3±1.74	81.39±5.83	125.77±3.37
**2-Pentylfuran**	**C3777693**	C9H14O	736.081	1.25304	12.36±2.08	19.2±1.73	37.41±1.59	59.8±3.37	16.05±0.23	69.93±3.61	108.33±2.68
**1-Methoxy-2-propylaceta**	**C108656**	C6H12O	729.044	1.14305	158.38±4.7	151.9±6.75	137.17±3.22	137.2±8.59	140.13±4.32	129.02±1.37	120.96±2.17
**(E)-2-Hexenal**	**C6728263**	C6H10O	716.376	1.18446	24.38±1.03	45.92±0.66	57.5±6.91	103.26±3.33	28.29±0.37	184.78±8.51	220.99±4.83
**3-Methyl-1-butanol-M**	**C123513**	C5H12O	702.302	1.24916	99.65±0.97	170.61±2.67	115.41±3.17	109.94±9.41	436.74±5.09	193.05±57.78	401.89±16.78
**3-Methyl-1-butanol-D**	**C123513**	C5H12O	702.302	1.49115	23.76±2.29	25.4±0.32	21.17±0.62	24.29±2.36	58.78±1.92	23.67±3.72	53.12±7.25
**Heptanal-M**	**C111717**	C7H14O	667.819	1.34751	472.88±48.06	1545.39±132.6	1918.02±46.81	2229.77±29.82	417.52±42.51	2248.67±94.25	1292.97±164.0
**Heptanal-D**	**C111717**	C7H14O	669.226	1.69433	78.42±2.89	549.58±118.7	969.17±80.41	1626.31±49.56	64.86±7.15	1719.06±211.26	398.92±127.32
**2-Heptanone**	**C110430**	C7H14O	662.189	1.26469	56.34±5.96	166.51±15.88	275.45±11.83	419.76±7.2	88.33±1.2	472.25±14.95	682.24±7.92
**1-Penten-3-ol**	**C616251**	C5H10O	632.632	0.94117	935.56±94.04	1893.4±42.63	2368.59±64.07	2948.79±16.15	1707.87±91.39	3315.07±25.42	3406.34±31.19
**2-Methyl-2-pentenal**	**C623369**	C6H10O	615.742	1.15728	11.22±0.67	16.38±1.25	17.25±0.97	34.13±1.76	11.36±0.49	55.88±4.12	85±3.81
**1-Butanol-M**	**C71363**	C4H10O	600.964	1.18446	261.18±15.94	384.19±2.09	471.2±40.79	687.64±14.73	580.12±9.14	769.95±34.43	895.57±17.4
**1-Butanol-D**	**C71363**	C4H10O	601.668	1.38892	17.14±1.14	27.82±1.12	41.43±5.88	94.93±1.61	50.47±1.62	122.39±8.55	165.03±3.24
**4-Heptanone**	**C123193**	C7H14O	598.149	1.21422	51.33±5.38	96.11±6.48	173.58±7.4	299.01±6.87	86.74±1.44	376.13±22.42	525.04±1.89
**(E)-2-Pentenal**	**C1576870**	C5H8O	578.445	1.10811	113.81±14.8	285.37±4.76	270.98±24.61	311.88±2.57	88.71±0.58	441.34±29.65	621.42±11.98
**Diallylsulfide**	**C592881**	C6H10S	566.481	1.12881	158.98±11.58	710.28±10.82	385.27±8.9	612.92±19.04	524.25±37.81	141.88±0.29	192.32±2.03
**2-Pentanol**	**C6032297**	C5H12O	548.888	1.22069	63.16±4.16	117.22±4.75	104.1±4.28	80.31±2.56	181.39±4.28	71.77±2.82	109.49±3.19
**beta-Pinene**	**C127913**	C10H16	529.183	1.20775	30.94±3.12	31.35±1.35	40.79±3.21	57.41±1.81	29.87±3.13	66.35±4.94	144.04±4.26
**3-Penten-2-one**	**C625332**	C5H8O	514.405	1.09258	52.78±3.56	50.05±1.58	42.78±2.13	46.59±4.64	46.62±1.92	83.67±4.54	124.53±7.43
**2-Methyl-1-propanol**	**C78831**	C4H10O	503.145	1.17669	253.67±3.55	255.05±12.6	156.72±1.42	94.14±3.47	436.24±16.26	123.06±22.32	272.6±22.34
**Hexanal-M**	**C66251**	C6H12O	489.07	1.28022	2972.19±312.9	5749.34±277.0	8268.66±225.89	10312.33±101.4	4248.73±279.2	9828±357.37	5975.77±450.9
**Hexanal-D**	**C66251**	C6H12O	490.478	1.56104	1458.18±358.2	5647.75±635.6	10310.16±603.2	16670.25±105.2	2983.78±389.1	14564.82±1471.6	5849.67±914.6
**1-Propanol-M**	**C71238**	C3H8O	428.137	1.11344	579.38±35.53	1194.53±32.99	2093.02±210.36	2957.12±82.12	1954.65±111.4	3174.84±45.94	3181.31±35.39

**1-Propanol-D**	**C71238**	C3H8O	428.137	1.25052	95.41±2.78	242.73±23.38	721.29±147.07	1965.61±250.46	495.03±60.06	4502.06±489.6	4335.96±120.4
**1-Penten-3-one-M**	**C1629589**	C5H8O	409.677	1.08171	98.56±8.06	217.36±3.21	229.52±8.2	199.03±1.71	57.57±1.74	182.78±4.69	222.84±12.14
**1-Penten-3-one-D**	**C1629589**	C5H8O	410.631	1.31057	16.19±1.54	28.26±2.22	32.89±1.99	36.17±1.32	12.19±0.69	36.5±3.15	45.61±4.93
**2-Butanol**	**C78922**	C4H10O	407.449	1.15535	122.41±5.99	75.5±1.69	67±1.43	83.75±2.49	74.38±2.01	125.76±5.52	140.21±5.78
**1**	**unidentifie**	*	393.444	1.14969	30.79±2.7	27.76±0.85	33.91±4.27	41.39±3.59	36.31±2.42	83.53±16.42	259.71±31.5
**2**	**unidentifie**	*	387.078	1.10777	125.01±1.76	174.27±2.33	193.26±2.46	169.01±6.59	104.03±0.87	280.7±14.13	361.13±2.07
**Pentanal-M**	**C110623**	C5H10O	364.799	1.2052	1259.36±87.7	1798.82±50.29	2098.32±8.98	2134.33±15.99	1030.37±127.7	1935.17±59.65	1444.48±21.28
**Pentanal-D**	**C110623**	C5H10O	365.117	1.42159	869.18±182.07	2767.79±227.4	4629.46±111.37	5365.54±224.42	587.62±152.67	3985.42±533.39	1294.19±288.2
**2-Pentanone**	**C107879**	C5H10O	363.844	1.35248	228.05±33.76	633.44±22.39	949.89±73.35	1672.2±62.74	430.14±2.98	2330.15±138.65	3404.34±10.68
**Propylacetate**	**C109604**	C5H10O	349.521	1.16668	17.3±2.87	34.86±1.99	72.27±6.07	117.72±6.64	19.07±0.83	138.86±7.07	161.8±3.43
**Ethylpropanoate**	**C105373**	C5H10O	344.428	1.13836	31.34±0.42	42.4±2.38	54.07±5.03	99.8±5.75	72.86±4.65	142.54±3.45	142.46±2.27
**Ethanol-M**	**C64175**	C2H6O	320.239	1.04093	1982.38±6.15	1941.21±8.55	1907.29±18.59	1743.91±6.3	1866.51±25.07	1573.29±31.33	1445.77±6.77
**Ethanol-D**	**C64175**	C2H6O	320.239	1.12816	4449.29±74.81	5055.79±15.56	5081.37±28.02	4682.2±27.93	6200.86±93.38	4740.25±98.31	5967.59±40.04
**2-Propanol**	**C67630**	C3H8O	313.555	1.09078	589.47±32.44	519.03±7.25	400.34±54.13	270.14±33.74	306.99±8.31	96.99±42.52	219.29±10.01
**3-Methylbutanal**	**C590863**	C5H10O	310.69	1.4012	620.8±89.77	826.61±35.17	1014.99±208.25	1754.72±234.17	1331.4±46.87	3444.69±524.82	1829.35±67.58
